# Mental deterioration of refugees and asylum seekers with uncertain legal status in Australia: Perceptions and responses of legal representatives

**DOI:** 10.1177/00207640231159297

**Published:** 2023-04-05

**Authors:** Mary Anne Kenny, Nicholas Procter, Carol Grech

**Affiliations:** 1 School of Law and Criminology, Murdoch University, Perth, Western Australia; 2 Clinical and Health Sciences, University of South Australia, Adelaide, South Australia, Australia; 3 Mental Health and Suicide Prevention Research and Education Group, Clinical and Health Sciences, University of South Australia, Adelaide; 4 University of South Australia, Adelaide, South Australia, Australia

**Keywords:** Refugees, asylum seekers, mental health, legal professionals, mixed methods research

## Abstract

**Background:**

Many developed countries have introduced strict measures designed to deter people seeking asylum. Measures such as held detention, insecure visas, restrictions work and services all impact the mental health of asylum seekers. In 2014 Australia introduced a ‘fast track assessment’ (FTA) system of processing refugee claims for asylum seekers who arrived by boat, those found to be refugees were only eligible for temporary residence. Legal professionals play a pivotal role in protecting the rights of asylum seekers and gain unique insight into the impact of the legal system has on clients mental health.

**Aim:**

To investigate how legal professionals in Australia perceived the impact of the FTA process on their clients.

**Methods:**

Mixed methods comprising of two phases – (i) an online survey and (ii) follow-up focus groups and interviews with legal professionals involved in assisting asylum seekers in the FTA process. An inductive thematic analysis was used to analyse the data.

**Results:**

Survey results were obtained from 38 legal professionals. Follow up in depth qualitative focus groups and interviews were conducted with 16 survey participants. The data demonstrate that legal professionals encounter clients in complex seemingly insurmountable mental health crises including deepening mental distress and deterioration, feelings of hopelessness, defeat and entrapment. Interviewees shared compelling examples of what they believed constituted a direct connection between asylum seekers experiencing uncertainty and deteriorating mental health over time with fluctuations in hopelessness, anger, withdrawal and suicidality. These negative impacts were often compounded by separation from family.

**Conclusions:**

The legal framework for determining whether an asylum seeker is a refugee can have a detrimental impact on the mental health of asylum seekers. The mental distress of asylum seekers and refugees is exacerbated by uncertainty linked to both delays in processing accompanied by sustained and ongoing uncertainty of legal status.

## Introduction

It is well documented that asylum seekers experience traumatic events and insecurity before and during the departure from their home country (Posselt et al., 2020). Post-displacement stressors significantly impact mental health (Procter, 2005, 2006; Sinnerbrink et al., 1997). Those seeking asylum after arrival in Western countries face challenges as they navigate the legal system (Posselt et al., 2020).

The United Nations Convention Relating to the Status of Refugees 1951 offers protection for individuals who fear persecution for reasons of ‘race, religion, nationality, membership of a particular social group or political opinion’.^1^ Securing legal status as a refugee normally means having access to a visa or permit which allows the person to remain lawfully in the receiving country and not be forced to return to their country of origin. Asylum seekers must navigate a complex legal and procedural process during refugee status determination (RSD). This includes explaining their fear of persecution and why they fear returning home.

Research has found that RSD procedures are linked directly to poor mental health status of asylum seekers (Bögner et al., 2010; Posselt et al., 2020). Deterioration in mental health is linked to administrative procedures where the timelines are open-ended or uncertain (Brekke, 2010) but also where deadlines are set causing a sudden movement of ‘frenzied time’ (Griffiths, 2014).

In Australia, lawyers and migration agents (collectively referred to as ‘legal professionals’) play a pivotal role in assisting asylum seekers navigate the RSD process. Legal professionals commonly encounter clients in distress (Graffin, 2019). They take detailed histories of their clients’ backgrounds (including exposure to traumatic events). Their unique role as advocates establishes relationships of trust. They are often able to identify whether clients are experiencing distress (Barrett et al., 2014). However, there is limited research on how legal professionals recognise mental health problems or how they manage such manifestations in their clients. One study in the United Kingdom (UK) found that legal professionals felt ill equipped to meet the needs of clients with mental health problems (Wilson-Shaw et al., 2012). Another study in the United States of America found lawyers working with unaccompanied refugee children had frequent concerns for the mental health of their clients and were often the only conduit for referrals to mental health services (Baily et al., 2014). Studies of lawyers in the UK revealed the process of providing legal assistance to asylum seekers was ‘emotional labour’ (Graffin, 2019; Westaby, 2010), not only due to documenting accounts of persecution, but also due to the impact of working within difficult environments including legal aid cuts, large caseloads and political enmity towards asylum seekers (Graffin, 2019).

Australian laws and policies have become increasingly complex over the last decade due to frequent reforms designed to reinforce strict border controls to deter people seeking asylum by maritime routes. An expedited procedure for RSD was introduced in 2014, known as ‘fast track assessment’ (FTA). This procedure applied to approximately 30,000 asylum seekers who arrived in Australia as ‘unauthorised maritime arrivals’ between August 2012 and December 2013. Those found to be refugees were only eligible for temporary visas with a very small number likely to obtain permanent residence. A new Labor government elected in May 2022 and has committed to ending the FTA process and to provide permanent residence for those on temporary protection or ‘safe haven’ visas (Australian Labor Party, 2021), however, these policies have yet to be implemented.

While the RSD process is supposed to be faster, in practice asylum seekers in the FTA caseload experience significant delays. On average it has taken up to 6 years for people to receive their first temporary visa (Kenny et al., 2022). Asylum seekers in this case load have also experienced held detention, denial of access to employment and support services (Australian Human Rights Commission, 2020; Khawaja & Stein, 2016). Several studies have shown that uncertainty around visa applications and visa status for this group causes pervasive anxiety (Fleay & Hartley, 2016; Khawaja & Stein, 2016; Van Kooy & Bowman, 2019).

While there has been previous research on the mental health of asylum seekers with insecure visa status (Newnham et al., 2019; Nickerson et al., 2019), there is no research that explores the impact of the FTA on the mental health of asylum seekers from the perspectives of legal professionals. There is some limited research on how legal professionals recognise mental distress; however, there have been no studies examining their response to distress of their clients beyond the issue of referrals to mental health professionals. Our study addresses this gap by examining the findings of a mixed-methods research study to investigate how legal professionals perceived the impact of the FTA system on the mental health of their clients. This paper reports the research findings of what legal professionals witnessed regarding the impact of the FTA system on the mental health of their clients. The findings in relation to the challenges legal professionals faced in responded to their clients will be the subject of a separate publication.

The current research with legal professionals sought to contribute to the literature on the inter-relationship between the mental health of clients and their legal needs. The specific aims of this study were to investigate how participating legal professionals were observing the mental distress in their clients and how legal professionals characterised the impact of the FTA process on the mental health of their clients. The study further aimed to understand how legal professionals responded to manifestations of distress experienced by their asylum seeking clients and their perceptions about the impact mental health distress had on the ability of their clients to participate in the legal process.

The rationale to focus on legal professionals to provide information about the mental health of the FTA caseload was based on several factors. First, legal professionals have a unique vantage point, they have experience with multiple clients and assisting them at different points across this particular legal process. Second, gathering information about traumatic events and assisting clients to navigate a complex system are specialised skills which require legal professionals to establish a relationship of trust and to understand the impact of mental health upon an asylum seeker’s ability to detail their claims.

## Method

### Study design

The study used mixed methods to investigate how legal professionals understood and responded to the mental distress of their clients. Legal professionals included both lawyers and registered migration agents. In Australia, migration agents can provide immigration advice and assistance (Office of the Migration Agents Registration Authority [OMARA], 2022).

The research comprised two phases – (i) an online survey and (ii) follow-up focus groups and interviews. The first phase involved a 19-question ‘Survey for Legal Professionals working with the Fast-Track caseload’ designed using Survey Monkey™ online software. The use of an online questionnaire survey tool was an appropriate way to reach legal professionals across Australia and allowed flexibility to complete at their convenience. Face validity of the survey tool was established by legal experts reading and providing feedback on the construction and clarity of the questions and the survey was piloted on a subset of intended participants.

The survey gathered basic demographic information (e.g. role, length of time assisting clients, number of clients) and included a mix of rating scale and open-ended questions prompting legal professionals to share their experiences with clients from the FTA caseload. Questions centred on how legal professionals identified and responded to the mental distress of their clients and how they perceived that the FTA process impacted their clients. The survey took approximately 20 to 30 min to complete.

The survey was anonymous^2^ and accessible from October 2017 to March 2018. Purposive and snowball sampling was used to recruit participants in the first phase of the study. Information about the study was shared through professional newsletters, professional conferences, legal aid organisations and community legal centres across Australia. Participants were invited to refer others to join the study.

Participants in the online survey were able to opt into being part of a follow-up focus group or interview. Contact details were collected separately so answers to the survey remained confidential. Those who completed the survey and volunteered for a focus group by a particular date went into a draw to receive one of two AUD$250 gift cards.

The second phase of the study used interviews and focus groups to further explore the research questions. Participants took part in a semi-structured discussion on their work with clients from the FTA caseload. One-on-one interviews were also held with participants who were unable to attend focus groups. All focus groups and interviews were led by the first author (MAK) except one which was led by the second author (NP) and audio recorded. Three of the four focus groups had an observer present to record non-verbal reactions and group interactions. Questions included: what do you see of the impact of the FTA system on the mental health of your clients? How do you respond to clients in distress? How does the client’s mental health impact the preparation of documents or applications? What do you see as the boundaries for legal professionals in terms of your responses to clients in mental distress? What suggestions of good practice have you adopted or learned from others?

Focus groups and interviews were conducted during August 2018–February 2019 in three major Australian cities and online. Four focus groups consisting of two participants were held in Melbourne, Sydney and one via Zoom video conferencing online. One focus group consisting of five participants was held in Perth. Three interviews were held with individual participants online via Zoom™ video conferencing.

### Participants

Inclusion criteria required participants (i) be a registered migration agent; (ii) have provided advice and assistance to asylum seekers in the FTA case load in the last 3 years and (iii) provided that assistance through a community legal centre, legal aid organisation and/or as part of a migration or legal practice. At the time of data collection, Australian law required lawyers to be registered as migration agents to give immigration advice (Wood, 2020), in 2021 the requirement for lawyers to be registered as migration agents ceased.

### Ethical considerations

Information regarding the study, participants’ rights and researchers’ contact details were provided on the survey and to participants in focus groups and interviews. The online survey contained a consent form completed by all participants. Participation was anonymous and voluntary; the survey did not ask participants to provide any identifying information about themselves or their clients. All participants were informed that while they may like to refer to examples of clients, we understood they were obliged to keep details confidential and they were asked not to disclose information which could lead to their identification or breach any client confidence. The study was approved by the human research ethics committees at both Murdoch University (#2022/052) and University of South Australia (#36721,#200664) in line with the National Statement on Ethical Conduct in Human Research (2007).

### Analyses

Survey responses were gathered on Survey Monkey™. Focus groups and interviews were transcribed verbatim. During the transcription process, all identifying information was removed. Recordings of interviews and focus groups were checked against the typed transcripts by the lead researcher (MAK) for accuracy prior to analysis.

A thematic analysis of the survey responses and transcripts was undertaken using the six-phase framework from Braun and Clarke (2006) thereby reporting meanings, experiences and the reality of participants. Step 1 involved data immersion. The first author, (MAK), read the entire data set, documenting and annotating early impressions. Step 2 involved generating initial codes using a theoretical thematic analysis; data that were relevant to the research questions was coded using NVivo™. There were no pre-set codes. The lead researcher worked systematically through the entire data set identifying significant aspects in the data that could form the basis of themes across the data set (Braun & Clarke, 2006). After the data were initially coded and collated, Step 3 involved sorting and collating the data into themes and sub themes; themes were generated inductively and without preconceived classifications. Themes and subthemes were vetted independently by the other researchers (NP and CG). Step 4 involved refining the themes and subthemes; some themes were removed where there was overlap or where there was not sufficient data to support them. At Step 5, themes were refined further to identify the story each theme told and consensus reached by the research team that themes were clear and comprehensive.

## Results

The online survey was accessed and commenced by 55 survey participants: 38 completed the entire survey, 30 participants provided contact details for focus groups, and 16 individuals participated in focus groups or interviews.

Data concerning the legal organisations where survey participants worked, the type of assistance provided, and the number of clients from the FTA caseload they had assisted are in Table 1. The sample was primarily female (68%). A large number (84%) of participants had assisted more than 20 clients from the FTA caseload in the last 3 years and over half (55%) had more than 5 years’ experience working with asylum seekers. Participants worked in private practice or community legal centres, some who worked in private practice also provided volunteer assistance. Participants provided a range of assistance to clients including support with visa applications, preparation of statements, attending interviews with the Department of Home Affairs, and general migration advice.

**Table 1.** Participant demographics – on-line survey.

Number of clients from the Fast-Track caseload assisted in the last 3 years *N* = 55	
1–10	6
11–20	7
More than 20	42
Years of experience working with asylum seekers and refugees *N* = 38	
Less than a year	1
At least 1 year but less than 3 years	7
At least 3 years but less than 5 years	9
At least 5 years but less than 10 years	12
10 years or more	9
*Capacity in which provided advice to clients n* *=* *38 (some participants worked in multiple capacities)*	
Volunteer/pro bono position with a community legal centre	16
Employee of a community legal centre or legal aid	16
Migration agent/Lawyer in private practice	18
Gender *n* = 38	
Male	12
Female	26

Data on the demographics of the participants in the focus groups and interviews are presented at Table 2. Participants came from workplaces across Australia. Most participants (69%) worked with asylum seekers in a community legal centre or voluntary capacity, around a third (31%) worked in private practice.

**Table 2.** Participant demographics – focus groups and interviews *n* = 16.

Location	
Victoria	6
New South Wales	3
Western Australia	5
South Australia	1
Australian Capital Territory	1
Gender	
Male	3
Female	13
Organisation type	
Employee of a community legal centre	7
Migration agent/Lawyer in private practice	5
Volunteer at pro bono clinic	4

All participants reported that in their work with clients from the FTA caseload they witnessed clients of emotional and/or mental distress. Four key themes were found: (1) ongoing uncertainty, (2) mental and physical deterioration, (3) lethal hopelessness and (4) suicidality. Verbatim quotes from participants are included under each theme to both illustrate and illuminate their experiences.

### Theme 1: Ongoing uncertainty

Participants reported that clients experienced uncertainty several years prior to lodging their protection claims – during flight from their home country, being held in immigration detention on arrival in Australia, and living in the community without work rights and insecure visa status.

The limbo that they’re in seems to be absolutely driving them mad. I’ve met clients at the beginning of their journey which is well probably the middle really, when they get to actually lodge their [visa] application, prepare it. So, they’ve already been waiting a few years and then I’ve come into contact with them at this point when they’re a few years in and finally able to tell their story. (Focus group participant (FGP) #6)

All those months, years they’ve had this story about what they’ve been through kind of pressing on them and no one’s interested, no one wants to know, no one cares and then suddenly after years of dealing with that trauma in whatever way they can, and sometimes it’s burying it or whatever, they have to suddenly kind of bring it all out. (FGP#3)

Uncertainty was pervasive. Even when clients were found to be refugees after the legal process, they were only eligible for a temporary visa, at the end of which they would have to be reassessed again and would only ever be eligible for another period of temporary stay. Participants witnessed clients with a deepening lack of hope, a never-ending state of mental distress.

Even for those that were granted a visa at the end that were owed protection and found to be refugees there was still so much uncertainty they still felt like they were in limbo, so many of them described to me that they were just kind of nowhere. (Interview participant (IP) #11)

I could see the lack of hope, the prolonged uncertainty, just the precariousness of their status here was having a much greater effect on their mental health (IP#15)

There is never going to be an end for them, they are always going to be worrying about the next application. whether they’re going to be able to stay. (FGP#4)

### Theme 2: Mental and physical deterioration

All focus group and interview participants reported witnessing clients crying during interviews.

Some clients avoid answering questions especially in relation to painful memories and may cry loudly and repeatedly. (Survey respondent (SR)#38)

All of my clients were men, and some just completely broke down [with] uncontrollable crying and weeping. (FGP#14)

Many attended appointments in a dishevelled state, malodourous due to poor hygiene, agitated, unable to sit still, shaking, sweating and collapsing. Others were clearly exhausted.

One participant described it this way.

An appointment is sort of trigger like a snap or a sort of a wake up and all this stuff that they sort of put off or try to block out all of a sudden kind of floods back in. (IP#9)

Another participant described an extreme physical reaction to stress.

One applicant was so affected by trauma that he would physically convulse on the floor if questioned. He had numerous neurological tests done and the only conclusion his medical team could come up with is that it was his body reacting to stress and trauma. I had never witnessed anything like it. (SR#55)

Mental deterioration and despair also featured.

It doesn’t matter how strong or resilient the clients are [the legal process] wears away at them, they’re made to feel at every step of the way that they’re less than. The delay has exponentially increased that feeling and I’m just seeing all of my clients’ nosedive now, mental health-wise. And I’m talking 200 cases I’ve got. (IP#16)

This latest process is at the end of other processes that may have extended over four or five previous years. It is this accumulated ‘experience’ means clients become despairing, ‘given up’, don’t care anymore. (SR#33)

### Theme 3: Lethal hopelessness

Hopelessness was identified as an expression of deteriorating mental health; feeling hopeless was mentioned by 15 participants (40%) in the online survey and featured strongly in each focus group and interview. This was related to the legal process (particularly delays), isolation, separation from family and feelings of burdensomeness.

Many clients clearly express symptoms of deteriorating mental health. . . say they are feeling hopeless. One client expressed ‘the department are killing me’ in reference to the long delays in processing his case. (SR#48).

I saw throughout the process there was more and more uncertainty, they started feeling quite vilified and I just saw them deteriorating . . . They started to feel guilty that they were here, they were saying the country doesn’t want me here, the community doesn’t want me here that’s why they’re making things so difficult. They didn’t want to ask for help, they didn’t want support because they felt like they weren’t entitled to it, that they were being punished for being here. (IP#11)

They see other families getting sponsorship and coming to Australia, that’s very difficult for them to explain and relate back to their families, particularly kids. (IP#14)

### Theme 4: Suicidality

Participants reported that many clients say they cannot continue and have nothing left to live for.

They talk about suicide and that they can’t take it but a lot of those people keep going because of their families. (FGP#5)

There’s probably been a couple of times where we’ve had to call ambulances or call a crisis mental health team and say hey this person’s you know right at the brink. (IP#10)

Many clients will tell me ‘It’s up to you now’ and/or ‘if I don’t get the visa you might as well kill me because I will not return to (X country)’ or will even say that they will suicide if they don’t get their visas. These comments are always very nihilistic and distressing. (SR#55)

One interview participant described a client died by suicide.

Unfortunately, we found out a few weeks later that he killed himself. So, he came to a country for safety and was completely let down. . .. Yeah, he never had a chance because he was just treated so poorly [he was] just completely defeated to the point of like giving up. (IP#10)

## Discussion

The aim of this paper was to explore how legal professionals perceived the impact of the FTA process on the mental health of their clients. The study revealed the unique vantage points lawyers and registered migration agents had garnered over several decades of collective experience with asylum seekers. The data demonstrate complex seemingly insurmountable crises, and the level of distress is exacerbated by processes of uncertainty linked to both delays in processing their claims intertwined with sustained and ongoing uncertainty of their legal status.

Individuals who arrive in a country seeking asylum know they must tell their story to authorities to gain recognition as a refugee. There are already challenges in communicating a narrative of persecution in a way which is credible due to trauma, language and culture (Kagen, 2003; Smith-Khan, 2017). With the FTA caseload, a key driver of distress was the inability to apply for a visa for several years, followed by a difficult, fast-paced and time sensitive application process in which they were expected to relay their story. Individuals felt marginalised and destabilised before the process had begun. Treatment during the process appeared to be just as, or even more, distressing than their narratives of trauma, a situation similar to findings reported elsewhere (Posselt et al., 2019). They were forced to suppress their stories and were then required to quickly relay their narrative leading to individuals experiencing a flooding of memories and traumatic experiences.

Physical and psychological safety is a widely regarded prerequisite for remembrance and recovery from trauma (Herman, 1998). In the case of the FTA caseload, the grant of temporary protection with no prospect of permanent settlement disrupts recovery. Even though many were legally accepted as refugees, they do not have certainty that they will not be returned to the country where they experienced trauma. Uncertainty and family separation combine with the open-endedness about their future undermine individual capacity to cope with everyday life and decision making. In the current study, legal professionals describe a trajectory of initial stress with beginning the process and continued deterioration throughout the process and after a visa is granted.

Participant’s description of client mental deterioration is consistent with a recent Australian study that found refugees and asylum seekers with insecure visa status had more severe symptoms of PTSD and depression compared to those on permanent visas (Byrow et al., 2022). This finding is supported by data from Germany revealing that insecure and temporary visa status contributes significantly to deterioration in mental health (Boettcher & Neuner, 2022; Winkler et al., 2018).

Ongoing states of uncertainty in the participants’ clients led to chronic and pervasive feelings of hopelessness. Legal practitioners described clients trapped by their insecure status and feeling a lack of control over their lives and future. Marginalised by a lack of acceptance and rejection from the Australian community meant their clients never belonged and occupied a status as ‘outlanders’. National government policy designed to penalise them based on their mode of arrival and was inextricably linked to feelings of burdensomeness and defeat. The lack of access to any permanent visa, having no way forward, underpins phenomena described as ‘lethal hopelessness’ (Procter et al., 2018).

Legal professionals are on the frontline working with clients who have and continue to suffer trauma. They hear stories involving overwhelming emotions (e.g. despair and anger) as well as witnessing self-harm and suicidal behaviour. Our findings extend previous research examining the emotional labour of legal representation of asylum seekers (Westaby, 2010) and vicarious trauma experienced by lawyers supporting asylum seekers (Piwowarczyk et al., 2009; Rønning et al., 2020). The current research, however, goes further. Our study is distinct from previous research as the legal professionals in in this study were not just asked to describe how they *responded* but were asked what they *observed*. In addition, the mental distress encountered by legal professionals went beyond clients’ responding to traumatic memories associated with describing their asylum claims but was clearly compounded by the FTA process and ongoing sustained uncertainty.

Our findings have significant practical implications for legal professionals who interview asylum seekers, frontline clinicians as well as for policy makers and researchers. One of the key implications of the current study is the rationale it provides for raising awareness about the compounding mental health impact of RSD/FTA on clients. Improving legal professionals’ understanding of this issue will enable them to make greater sense of the experience of their clients, respond better and to consider alternative models of work (such as holistic or combined service with targeted mental health supports). Finally, policy makers should end the uncertainty caused by temporary protection and allow refugees access to permanent visas (Kenny et al., 2022). The new Labor government has already made a commitment for positive change offering some hope for this marginalised group (Martin, 2022).

### Strengths and limitations

The findings should be interpreted in the context of study strengths and limitations. The strengths of this study include the detailed comprehensiveness of the interview and survey data collected, the specific timing of data collection (i.e. from legal professionals during RSD), and the rigorous processes of data analysis. The first author has extensive previous experience conducting interviews with asylum-seeker applicants, refugee and asylum-seeker law and an in-depth knowledge of trauma-related distress in this population. Study limitations include a cross-sectional design and a relatively small sample size, possibly due to the length of the original survey instrument which was necessary to gain a comprehensive understanding of participants’ views. Finally, this study relied exclusively on data from legal professionals. Future research could seek additional data from asylum seeker participants.

## Recommendations for further research

Given the ongoing intensity of drivers of distress, future research is needed to understand the precise nature of helpful approaches and interventions to mitigate critical moments of mental suffering and deterioration. The progression of an unendurable and insurmountable worsening trajectory towards a mental health crisis can be catastrophic as it can fast moving. It can involve a full spectrum of thoughts and behaviour, feeling a burden to oneself and to others, and a deep sense of hopelessness and suicidality. Fluctuating experiences of distress for refugees and asylum seekers accompany moments of being able to ‘get through’ and reasons to remain strong. Precisely how to mitigate and prevent distress within such scenarios is unclear. Further research could also shed light on how legal professionals themselves prepare and cope with difficult conversations and witnessing of distress. Larger sample sizes and a longer-term follow-up should feature in a study design.

## Conclusion

As governments around the world develop policies for protection for people seeking asylum, our study reports on the experiences of 38 legal professionals relating to the mental health of asylum seekers with insecure visa status in the Australian community. A rigorous inductive thematic analysis revealed reports of deepening mental distress and deterioration, feelings of hopelessness, defeat and entrapment. Legal professionals shared compelling examples of what they believed constituted a direct connection between asylum seekers experiencing uncertainty and deteriorating mental health over time with fluctuations in hopelessness, anger, withdrawal and suicidality. The troubles and difficulties experienced by asylum-seeker clients were made worse by separation from family. Durable settlement solutions and clear timelines for refugee status resolution, combined with mental health support for legal professionals and their clients, will likely go a long way to prevent future mental distress and deterioration. Findings have implications for community-based health and social care providers, legal professionals and government policy makers.

## Notes

1. Article 1A

2. Note that participants were advised that as the data were collected through Survey Money, a United States of America platform, the United States government, under US privacy laws, has the right to access all information held in electronic databases.
